# Assessment of the Status of Awareness, Ownership, and Usage of Long-Lasting Insecticide Treated Nets after Mass Distribution in Ekiti State, Nigeria

**DOI:** 10.1155/2019/1273714

**Published:** 2019-04-01

**Authors:** Adetunji Omonijo, Adejumoke O. Omonijo

**Affiliations:** ^1^Department of Family Medicine, Federal Teaching Hospital, Ido Ekiti, Ekiti State, Nigeria; ^2^Department of Animal and Environmental Biology, Federal University Oye Ekiti State, Nigeria

## Abstract

Vector control with long-lasting insecticide treated nets (LLINs) has been identified as a major component of malaria prevention and control. The study examined present status of awareness, ownership, and utilization of LLINs in malaria high-risk areas of Ekiti State, Nigeria. Data were obtained from 352 copies of semistructured interviewer-guided questionnaire distributed to participants of each household in the four Local Government Areas (LGAs) of Ekiti State, where malaria is endemic after mass distribution of LLINs. Findings in this study showed that awareness was high (91.8%) in the Local Government Areas (LGAs) with mass media contributing largely (44.3%) to awareness. Also, LLINs ownership was found to be high (71.3%) with 72.9% of the nets being supplied by the government. Of the owners of LLINs, usage rate was observed to be 67.6%. Multivariate analysis result showed that statistically significant sociodemographic characteristics of respondents predicting the usage of LLINs included age greater than 50 years (p value = 0.008), female gender (*χ*^2^ = 8.2014, p value = 0.004), being married (*χ*^2^ = 24.721, p value <0.001), civil servants (*χ*^2^ = 12.739, p value = 0.005), and average income above poverty line (*χ*^2^ = 13.576, p value = 0.004). The study concluded that although not all households surveyed owned LLINs, nevertheless, the level of usage of LLINs among net-owning households was high. The study recommended continuous free distribution, periodic household survey, and expanding public knowledge on the benefits of LLINs usage especially through social media.

## 1. Introduction

Malaria remains a horrendous disease constituting a global burden. Malaria was responsible for an estimated 219 million cases and 435 000 deaths globally [1]. In Nigeria, according to the National Malaria Strategic Plan (2014-2020), malaria is responsible for 60% of outpatient visits to health facilities, 30% of childhood death, 25% of death in children below one year of age, and 11% of maternal death [2, 3]. The economic impact of the disease in Nigerian households is evident from huge financial resources that are involved in control and treatment, absenteeism at work, and disability adjusted life years (DALYs), thereby causing significant drawback to socioeconomic growth [4]. Synergetic efforts through vector surveillance approach, educational campaigns, and wide distribution of long-lasting insecticidal nets (LLINs) have successfully reduced malaria burden in endemic regions. Among several interventions, long-lasting insecticidal nets (LLINs) have played an important role in reducing the global malaria burden since 2000 [5]. Evaluation of LLINs success over years has resulted in treatment modification of nets due to the development of insecticide-resistance by malaria vectors, thereby leading to pyrethroid-PBO nets being given an interim endorsement as a new WHO class of vector control products [3]. Although LLINs are a key tool used widely by people at risk of malaria, some communities have not been able to translate the available malaria control interventions to effective opportunities to curtail the disease. Also, there is evidence that relatively few people in endemic regions access and use LLINs (Ibor et al., 2012).

Studies have shown the importance of regular assessment of LLINs distribution in identifying shortcomings and creating a roadmap for future success such as mapping behaviour change communication (BCC) activities by all partners and stakeholders as well as a secondary analysis of existing data from postcampaign surveys [6]. Hence, this study was designed to provide an update on LLINs awareness, ownership, and usage in Ekiti State.

Ekiti State is one of the Southwestern States which is associated with high incidence of malaria disease among youths in all the LGAs [7]. The state is one of the beneficiaries of wide LLINs distribution through Global Fund in year 2010 [8, 9].

A LLIN post-distribution-survey carried out in the state in 2014 showed that the level of LLINs utilization was 58.5%. This was far below the World Health Organization target of 100% [10]. The aim of this research is to assess the present status of LLINs' ownership and usage among the people of Ekiti State and how it translates to the wellness of vast population of Ekiti populace with the view of incorporating the pro- or antisustainability into planning towards malaria eradication in the nearest future.

## 2. Materials and Methods

### 2.1. Study Area

The study was carried out in four Local Governments Areas, namely, Ado Local Government Area, Ido/Osi LGA, Ikole, and Oye LGAs. The state is in one of the three major malaria epidemiological zones in Nigeria and is characterized with tropical climate with alternating rainy season (April-October) and dry season (November-March). Temperature ranges between 21°C and 28°C with high humidity, all favorable to malaria vectors development.

### 2.2. Sample Size Calculation

Sample size was calculated using the equation n=1.96^2^ pq/L^2^, where n represents sample size, p is expected prevalence (0.5), q=1-p (1-0.5), and L denotes limits.

### 2.3. Study Design/Sampling Method

**Figure 1:**
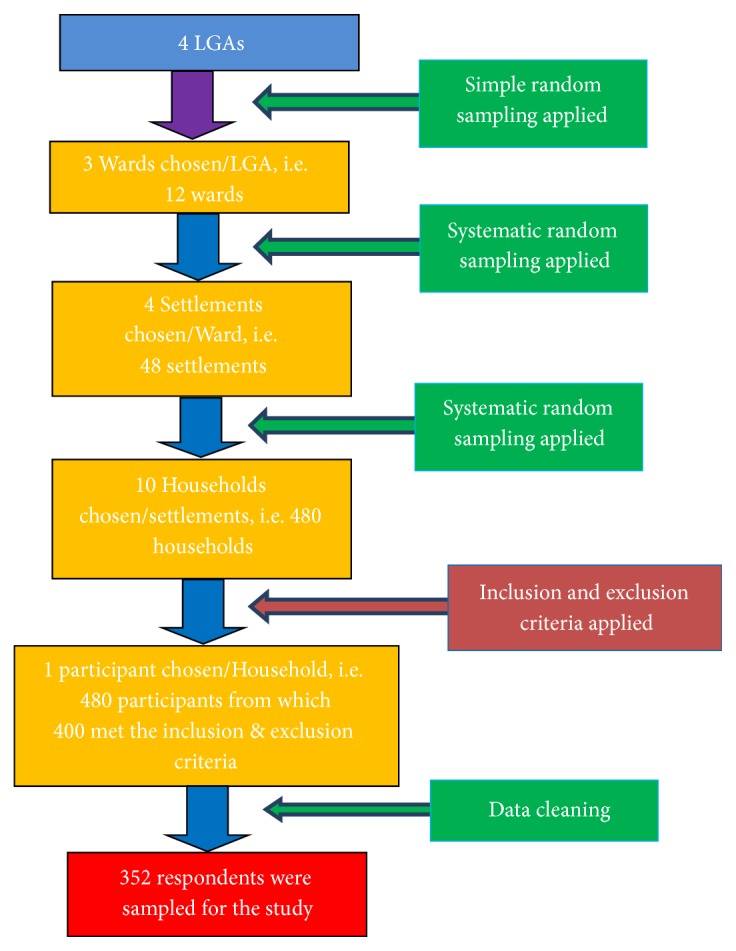
Study flowchart.

A descriptive cross-sectional study was used in this study. Multistage sampling technique was used in view of the large size of the study area. All the four LGAs in the study area were included in the study. Simple random sampling technique was used in selecting 3 wards from each LGA. At the ward level, systematic random sampling method was used to select four settlements from the lists of all the settlements in each ward. Finally, ten households were selected by systematic random sampling method from a line list of all households in each settlement to participate in the survey. A total of 480 participants from the households were chosen, out of which 400 participants met the inclusion and exclusion criteria. Finally, after data cleaning, 352 participants from the households spread across the 4 LGAs in the state were used for this study.  Figure 1 showed the flow-chart diagrams used in the study design.

### 2.4. Data Collection/Questionnaire Administration

A total of 400 semistructured interviewer-guided questionnaires were administered to those who met the inclusion and exclusion criteria to obtain information on sociodemographic characteristics, awareness, ownership, sources and usage of LLINs, sleeping patterns, and frequency of malaria attacks among other information. Questionnaires were pretested in Moba Local Government Area of Ekiti State.

Data collection lasted for two months between August and October 2016 during the raining season. Trained interviewers were employed. The interviews were conducted in English or Yoruba languages. The interviewers were frequently supervised on the field by the principal researcher to monitor data collection and provide necessary feedback. Each respondent was interviewed for 20 minutes. Appropriate community entry was done through the community leaders.

### 2.5. Statistical Analysis

All data collected were analyzed using the Statistical Package for Social Sciences (SPSS) for Windows software (version 20) (SPSS Inc., Chicago, IL, USA). The data were presented in tabular form, frequency tables were generated for relevant variables, and percentages were determined as appropriate. Pearson's chi-square test was used to assess the bivariate association between awareness, ownership, and usage of LLINs with the respondents' sociodemographic characteristics. P value equal to or less than 0.05 was considered as statistically significant.

### 2.6. Ethics Approval and Consent to Participate

Ethical approval was waived as there were no sensitive issues mentioned in the questionnaire. Participation was voluntary. Confidentiality of the information was assured and maintained by using an anonymous process.

## 3. Results

A total of 352 respondents were involved in the study.  Table 1 showed the sociodemographic characteristics of the respondents. The table showed that more than half (59.1%) of those who use LLINs were females. The age distribution of persons sleeping under LLINs showed that highest users of LLINs (73.6%) were between 18 and 30 years of age, while lowest users of LLINs (1.7%) were ≥50 years of age. It was observed that majority (87.8%) of the respondents were Christians. Yoruba ethnicity ranked highest (81.3%) among the respondents. Civil servants and students represented a larger percentage, 40.3% and 36.5%, respectively, while others were self-employed (19.0%) and farmers (4.3%). Majority (52.2%) of those who slept under LLINs earned a monthly income ≥$57.

Table 2 showed the assessment of the level of awareness, ownership, and usage of LLINs among respondents. Findings in this study showed that awareness was high (91.8%) in the LGAs with mass media contributing largely (44.3%) to awareness followed by friends (26.5%), Internet (18.3%), and books (11.1%). Also, LLINs ownership was found to be high (71.3%) with 72.9% of the nets being supplied by the government. Of the 71.3% of owners of LLINs, usage rate was observed to be 67.9%, out of which more than three-quarters of respondents (81.9%) sleep under a net every night.

Table 3 showed the assessment of knowledge of malaria transmission, sleeping pattern, and frequency of malaria attacks among respondents. Majority of the respondents have good knowledge of malaria transmission (96.3%), sleep in open room (90.6%), and used beds as sleeping materials (91.5%). Also, more than half (57.1%) of the respondents reported frequent episodes of malaria attacks.

Table 4 showed the relationships between usage of LLINs and sociodemographic characteristics of respondents. It was observed that sociodemographic characteristics of respondents predicting the usage of LLINs that were statistically significant included age greater than 50 years (p value = 0.008), female gender (*χ*^2^ = 8.2014, p value = 0.004), being married (*χ*^2^ = 24.721, p value <0.001), civil servants (*χ*^2^ = 12.739, p value = 0.005), and average income above poverty line (*χ*^2^ = 13.576, p value = 0.004).

## 4. Discussion

### 4.1. Level of Awareness of LLINs among Respondents

The outcome of awareness of LLIN in this study is in agreement with 95.3% obtained in Ekiti State in mass predistribution campaign in 2014 [10] and 97.9% reported from Oyo state. Similar trend was also reported from regions outside Nigeria [11, 12]. This corroborates the impact of campaign on LLINs coverage and the need to maintain such strategy in sustaining the progress recorded [13]. The trend observed in the contribution of campaign strategies to LLINs awareness is in agreement with the result from behaviour change communication survey reported by [6, 14].

Out of all the campaign strategies, mass media (44.3%) remains the most important means of creating awareness as observed in this study followed by friends (26.5%), Internet (18.3%), and books (11.1%). This is in agreement with the result from behaviour change communication survey reported by [6, 14].

### 4.2. Level of Ownership of LLINs among Respondents

The high ownership level that was observed in this study was in agreement with the record of 72.6% reported in a study done in Ethiopia [15]. It was, however, lower than 95.3% that was reported previously in the state in 2014 [10]. Also, the role of government in the increased coverage rate cannot be overemphasized as most of the acquired nets were supplied by the government. This is in agreement with the established report of [15] which associated increased LLINs ownership recorded in Rwanda with government contribution.

### 4.3. Level of Usage of LLINs among Respondents

The level of usage observed in this study agrees with reports from other authors. For instance, 63% was reported by [6] from postcampaign studies in Nigeria, 76.5% usage was reported in Sierra Leone [16], 68.3% in Togo, 65% in Ethiopia, 72% in Rwanda [15], and 81% in India [17]. However, a low percentage of usage of LLIN (21.7%) was observed in a study conducted in Cross River. Reasons given for low level of usage included lack of awareness of LLIN, nonownership of the nets, high cost of ITNs, and alternative malaria prevention and mosquito control other than ITNs (Ibor et al., 2012).

Although the usage percentage (67.6%) observed in this study is higher than the 58.5% reported in 2014 [10], it is still far from 100% global utilization target. The increased usage could be linked to training and education provided to LLINs owners on usage. This is in conformity with the result that was reported in Sierra Leone [16, 18].

Furthermore, the high percentage (91.5%) of people sleeping on bed recorded in this study is far higher than that reported in East Rwanda (62.9%) [15]. This perhaps partly contributed to the increased usage observed in the study area as supported by reports from other studies [15] that associated not sleeping on a bed with nonusage of LLINs.

### 4.4. Assessment of Knowledge of Malaria Transmission, Sleep Pattern, and Frequency of Malaria Attacks among Respondents

Studies have shown that ownership and usage of LLINs affect vector population survival and offer protection to those not sleeping under it, thereby achieving mass protection [19]. In this study, however, despite an appreciable increase in usage with 96.3% having proper knowledge of malaria transmission, malaria attacks were frequently recorded in over 50% of the respondents. This is probably due to inconsistent usage (18.1%) and sleeping pattern observed among LLINs users. Majority (68.2%) of LLINs users were observed to stay outdoor between the hours of 19.00 and 23.00. This possibly exposed them to mosquito bites before sleeping under mosquito nets, thereby hampering the overall effectiveness of mosquito nets.

Several studies have reported similar trend in outdoor malaria transmission. For instance, 36.4% was reported in the west of Eritrea [20] and 49% in Uganda [21]. Although most of these reports were made before wide distribution of LLINs, studies have shown that high coverage of LLINs resulted in a biting shift pattern in some species of mosquito vectors in some regions [22–26] which led to persistence outdoor residual transmission [27]. Evidently, malaria elimination requires a combination of interventions [28]. Multiple interventions such as larval source management, indoor residual spraying, and mosquito repellents have been widely and successfully used as complementary tools to LLINs in eliminating malaria vectors [27, 29, 30]. While LLINs usage still remains a pivotal control measure in curtailing malaria irrespective of resistance development [31], awareness should be created on personal protection through behaviour change communication activities.

## 5. Conclusion

The current study evidently showed the importance of continuous free distribution, periodic household, and continuous education via mass media on sustainability of LLINs ownership and usage in the study area. Efforts should not be relented on continuous use of these tools in regions where increased ownership and usage have been recorded. Also, owners of LLINs should be educated on proper and correct usage of the nets. Although the awareness and usage of LLINs are high in the study area, there is uneven utilization among socioeconomic groups; hence, free distribution of LLINs to increase household ownership could be a catalyst to increased open equitable usage across age groups and gender.

Furthermore, while it may be difficult to divulge people from the habits of staying outdoor in the evening in an African setting, awareness can be intensified on the total avoidance of mosquitoes through behavior change communication interventions and use of long-lasting insecticide treated nets.

## Figures and Tables

**Table:** (a) Sociodemographic characteristics of the respondents

Variables	N = 352	Percentage (%)
*Age*		
18 – 30	259	73.6
31 – 40	38	10.8
41 – 50	49	13.9
>50	6	1.7
*Gender*		
Male	144	40.9
Female	208	59.1
*Education level*		
None	2	0.6
Primary	12	3.4
Secondary	73	20.7
Tertiary	265	75.3
*Marital status*		
Single	160	45.5
Married	187	53.1
Divorced	5	1.4

**Table:** (b) Sociodemographic characteristics of the respondents

Variables	N = 352	Percentage (%)
*Religion*		
Christianity	309	87.8
Islam	36	10.2
Traditionalists	7	2.0
*Ethnicity*		
Yoruba	286	81.3
Igbo	25	7.1
Hausa	12	3.4
Ebira and others	29	8.2
*Occupation*		
Civil servants	142	40.3
Self employed	67	19.0
Farmers	15	4.3
Students	128	36.5
*Average Income*		
<Poverty Line (<*$57*)	168	47.8
≥Poverty Line (>*$57*)	184	52.2

$57 is the expected minimum income per month equivalent of $1.90 per day. (Poverty/Data [Internet]. Data.worldbank.org.2017).

**Table:** (a) Assessment of the level of awareness, ownership, and usage of LLINs among respondents

Variables	N = 352	Percentage (%)
*Awareness*		
Yes	323	91.8
No	29	8.2
*Source of information on LLINs (n=323)*		
Mass media	143	44.3
Friends	85	26.3
Book	36	11.1
Internet	59	18.3
*Ownership*		
Yes	251	71.3
No	101	28.7
*Sources of LLINs (n=251)*		
Government	183	72.9
NGO	19	7.6
Purchase in Pharmaceutical shop	39	15.5
Others	10	4.0

**Table:** (b) Assessment of the level of awareness, ownership, and usage of LLINs among respondents

Variables	N = 352	Percentage (%)
*Reasons for not owning LLINs (n=101)*		
Lack of information	22	21.8
High cost of LLINs	12	11.9
Not distributed by the Government	43	42.6
Use of alternative malaria prevention	24	23.7
*Education on usage*		
Yes	239	67.9
No	113	32.1
*Usage*		
Yes	238	67.6
No	114	32.4
*Usage frequency*		
Every night	195	81.9
Occasionally	43	18.1

**Table:** (c) Assessment of the level of awareness, ownership, and usage of LLINs among respondents

Variables	N = 352	Percentage (%)
*Reasons for non-utilizing of LLINs (n=114)*		
Cause of heat	22	19.3
Net too small	19	16.7
Disturbs sleep	12	10.5
Cultural belief	19	16.7
LLINs not distributed	26	22.8
Tacking problem	16	14.0

**Table 3:** Assessment of knowledge of malaria transmission, sleep pattern, and frequency of malaria attacks among respondents.

Variables	N = 352	Percentage (%)
*Staying outdoors*		
Stay outdoors between 19.00 and 23.00 hr	240	68.2
Do not	112	31.8
*Materials for Sleeping*		
Mats	30	8.4
Beds	322	91.6
*Sleeping place*		
Open room	33	9.4
Bed room	319	90.6
*Proper knowledge of malaria transmission*		
Yes	339	96.3
No	13	3.7
*Frequency of Malaria attacks*		
Frequently	201	57.1
Occasionally	151	42.9

**Table 4:** Relationships between usage of LLINs and sociodemographic characteristics of respondents.

Usage of Insecticide Treated Net				
Variables	Yes	No	*χ* ^2^	P value
	n=238 (%)	n = 114 (%)		
*Age*				
18 – 30	176 (68.0)	83 (32.0)		***0.008*** ^**∗**^
31 – 40	19 (50.0)	19 (50.0)		
41 – 50	37 (75.5)	12 (24.5)		
>50	6 (100.0)	0 (0.0)		
*Gender*				
Male	85 (59.0)	59 (41.0)	8.204	***0.004***
Female	153 (73.6)	55 (26.4)		
*Education level*				
None	2 (100.0)	0 (0.0)		0.565^**∗**^
Primary	7 (58.3)	5 (41.7)		
Secondary	49 (67.1)	24 (32.9)		
Tertiary	180 (67.9)	85 (32.1)		
*Marital status*				
Single	88 (55.0)	72 (45.0)	24.721	***<0.001***
Married	148 (79.1)	39 (20.9)		
Divorced	2 (40.0)	3 (60.0)		
*Religion*				
Christianity	207 (67.0)	102 (33.0)		0.515^**∗**^
Islam	27 (75.0)	9 (25.0)		
Traditionalists	4 (57.1)	3 (42.9)		
*Ethnicity*				
Yoruba	199 (69.6)	87 (30.4)		0.390^**∗**^
Igbo	14 (56.0)	11 (44.0)		
Hausa	8 (66.7)	4 (33.3)		
Ebira and others	17 (58.6)	12 (41.4)		
*Occupation*				
Civil servants	111 (78.2)	31 (21.8)	12.739	***0.005***
Self employed	9 (60.0)	6 (40.0)		
Farmers	43 (64.2)	24 (35.8)		
Students	75 (58.6)	53 (41.4)		
*Average Income*			13.576	***0.004***
<Poverty Line (<$57)	102 (60.7)	66 (39.3)		
≥Poverty Line (≥$57)	136 (73.9)	48 (26.1)		

^*∗*^Fisher's exact test applied.

$5 is the expected minimum income per month equivalent of $1.90 per day. (Poverty/Data [Internet]. Data.worldbank.org.2017).

## Data Availability

The research data used to support the findings of this study are available from the corresponding author upon request.

## Additional Points

*Study Limitations.* The study was conducted in only four LGAs of Ekiti State and therefore results may not be generalized to the country. The study was only based on interviews and there were no observations conducted to validate the reported use of mosquito nets.
